# Associations of chronic illnesses and socio-demographic factors with health-related quality of life of older adults in Nigeria: A cross-sectional study

**DOI:** 10.4314/gmj.v54i3.7

**Published:** 2020-09

**Authors:** Joel O Faronbi, Aishat O Ajadi, Robbert J Gobbens

**Affiliations:** 1 Department of Nursing Science, College of Health Science, Obafemi Awolowo University, Ile-Ife, Nigeria; 2 Frail Elderly Research Support Group (FRESH), Institute of Neuroscience and Physiology, Sahlgrenska Academy, Gothenburg University, Gothenburg, Sweden; 3 Faculty of Health, Sports and Social Work, Inholland University of Applied Sciences, Amsterdam, the Netherlands; 4 Zonnehuisgroep Amstelland, Amstelveen, the Netherlands; 5 Department of General Practices, University of Antwerp, Belgium

**Keywords:** Chronic illness, Health-Related Quality of Life, Older adults, Socio-demographic factors

## Abstract

**Background:**

The increase in life expectancy has brought about a higher prevalence of chronic illnesses among older people.

**Objectives:**

To identify common chronic illnesses among older adults, to examine the influence of such conditions on their Health-Related Quality of Life (HRQoL), and to determine factors predicting their HRQoL.

**Method:**

A population-based cross-sectional study was conducted involving 377 individuals aged 60 years and above who were selected using multi-stage sampling techniques in Olorunda Local Government, Osun State, Nigeria. Data were collected using an interviewer-administered questionnaire comprising socio-demographic characteristics, chronic illnesses, and the World Health Organization quality of life instrument (WHOQOL-BREF) containing physical health, psychological, social relationships, and environmental domains.

**Results:**

About half (51.5%) of the respondents reported at least one chronic illness which has lasted for 1–5 years (43.3%). The prevalence of hypertension was 36.1%, diabetes 13.9% and arthritis 13.4%. Respondents with chronic illness had significantly lower HRQoL overall and in the physical health, social relationships and the environmental domains (all p<0.05) compared to those without a chronic illness. Factors that predicted HRQoL include age, marital status, level of education, the presence of chronic illness and prognosis of the condition.

**Conclusion:**

This study concluded that chronic illness is prevalent in Nigerian older people and significantly influence their HRQoL. Age, marital status, and level of education were associated with HRQoL in this group.

**Funding:**

Postdoctoral fellowship from Consortium for Advanced Research Training in Afric

## Introduction

The population of older people is increasing in all countries of the world. A great majority (two-thirds) of those over 60 years old live in developing countries, and this proportion is increasing steadily and will reach nearly three-quarters by the 2030s.^1^

However, in a country like Nigeria, where the inability of the government to cope with regular payment of pensions to the retired workforce; living in environments with weak health system as well as an acute lack of geriatrics care, is posing an enormous challenge to the health of older people.^2^ Chronic illnesses are major health concerns in both developing and developed nations.^3^

They are a leading cause of mortality in the world, representing 63% of all deaths, 29 million deaths worldwide.^4^ For a developed nation, chronic illnesses are known to be influenced by lifestyle changes, the effect of urbanisation, and diet^5^, while in a developing nation, it may be a result of malnutrition and repeated infection.

Even though the cause and frequency of chronic illnesses may be similar in countries with different economic levels, its impact on the population may not be the same. The impact is worse on developing nations than in the developed, probably due to limited access to health services and technologies for prompt detection, treatment, and management of cases.

People are more able to cope well and live with chronic illnesses in developed nations than in developing countries. Besides, some illnesses that may progress into chronicity are well controlled or eliminated in high-income countries, thus reducing the overall population burden.

Chronic illnesses occur in more than half of the American population.^6^ A similar finding is observed among the Chinese population and the prevalence increases with age^7^ Peltzer and Phaswana-Mafuya^8^ also reported that about 50% of the South African population had one chronic noncommunicable disease (NCDs) and that the most prevalent were hypertension and arthritis. In Nigeria, a study conducted by Abdulraheem *et al*, also reported the occurrence of chronic illness in 64.9% of the population.^9^ This finding confirms the assertion that chronic illnesses are present in both developed and developing nations and it is even more present in Nigeria.

Schultz and Kopec^10^ argued that chronic illnesses often result in lower overall health, physical health, mental well-being and the ability to function independently. The consequences may vary considerably depending on the specific condition. Individuals with chronic conditions often tend to have declined health-related quality of life (HRQoL), and the situation may be worse when there is multimorbidity.^11^–^13^ Furthermore, a previous study has shown that the greater the number of comorbidities, the worse the HRQoL in older age.^14^

Previous studies on the HRQoL of older people in Africa have been conducted mostly within hospitals with a focus on a specific disease condition.^15^–^17^ Only a few studies concerning this topic have been carried out at the community level on this continent. These include studies that examined the HRQoL among allergic rhinitis patients^18^, patients with tuberculosis^19^, adolescents living with sickle cell disease,^20^, and oral HRQoL of school teachers.^21^ However, none of these studies investigated the impact of specific illnesses on the HRQoL of older people in Nigeria. In this study, we report the common health problems among older adults in Olorunda Local Government, their HRQoL and identify major contributing factors to HRQoL. This will, therefore, contribute to interventions aimed at improving the health and wellbeing of the older adults' population locally and internationally. The objectives are to (i) identify the common chronic illnesses among the older adults in Olorunda Local Government, Nigeria; (ii) examine the influence of these conditions on the HRQoL of the older adults; and (iii) determine the associations between socio-demographic factors, chronic illnesses, and HRQoL.

## Methods

### Study population and data collection

A cross-sectional study was designed to assess the HRQoL of the older adults in Osogbo, Osun State, Nigeria. The indigenous Osun people predominantly occupy the city. The city has two local governments namely Olorunda and Osogbo, with Oloriunda comprising 11 wards and 131,649 residents.^22^

The target population included all adults 60 years of age and above, residing in the city for at least one year before the study. This was to ensure that data were collected from only regular inhabitants of the city, ensuring the enrolment of stable residents. A multi-stage sampling technique was used to select participants. Three wards were randomly selected using a town map obtained from the National Population Commissions. All streets in each of the selected wards were enumerated, and three streets were randomly selected using the table of random number. The selected streets were visited, and all households mapped out, giving approximately 40,000 households.

Households were randomly selected using a systematic sampling technique, and eligible older adults included in the study. Inclusion criteria for the study were older adults (aged 60 years and over), residents in the study area and cognitively intact. In case of more than one eligible participant in the dwelling, we enrolled only one by lottery method.

The study sample size was estimated using the formula: n = p (1-p) z^2^/D^2^. Where n= sample size; P = prevalence rate of chronic illnesses estimated at 50%, Z= standard normal variance where confidence level is 1.96 at 95%; D= absolute precision or error margin (5%).^23^ Based on a prevalence rate of chronic illness of 50%^24^ and 5% attrition rate, the sample size was estimated to be 400, to be selected proportionately to the size of the selected wards.

Ethical clearance was obtained from the Human Research Ethics Committee, Institute of Public Health, Obafemi Awolowo University, Ile-Ife, Nigeria (IPHOAU/12/101). Permission to collect data was obtained from the administrative head of the local governments. Older adults that consented were administered the questionnaire, including questions about socio-demographic factors, chronic illnesses, and the WHOQOL-BREF.

Data collection took six weeks (between October and November 2015). The instrument was administered to 400 older adults; however, only 377 participants (94%) who had complete data on selected variables were included in the analysis of this study. Data were collected after pilot testing and training by the first and second authors.

### Measurements

#### WHOQOL-BREF

HRQoL was assessed by the WHOQOL-BREF, an international cross-culturally comparable HRQoL instrument which determines the individual's perceptions in the context of their culture and value systems, and their personal goals, standards, and concern.^25^ The WHOQOL-BREF contains 26 items, with the first two assessing the overall quality of life and general health, respectively. The two questions were rated on a five-point Likert scale, where 1 = very poor and 5= very good and 1= very dissatisfied, and 5 =very satisfied respectively. The WHOQOL-BREF measures physical health (7 items); psychological (6 items); social relationships (3 items); and environmental domains (8 items), on a five-point scale ranging from 1 to 5. The overall (total) HRQoL was computed by summing up all the items on the list.

The total HRQoL was then transformed to give a score ranging from 0 to 100. In this study, we compared those with good HRQoL versus poor HRQoL with the view of establishing the impact of chronic illnesses on the HRQoL as measured by poor versus good HRQoL. Domain scores of the WHOQOL-BREF correlates highly (0.89 or above) with WHOQOL-100 domain scores^25^–^27^ from which it is derived. The instrument was translated into the local language (Yoruba) by an expert from the Department of Linguistics, Obafemi Awolowo University, Ile-Ife, Nigeria and pilot tested among twenty older adults in a different but similar setting. The Cronbach's alpha for the overall HRQoL was 0.767 and for the domain's physical health, psychological, social relationships, and environmental the Cronbach's alpha was .702, .685, .593, and .697, respectively. This is consistent with other previous studies using a similar setting.^28^, ^29^ It was therefore deemed suitable for use in the current study.

### Socio-demographic characteristics and chronic illnesses

Socio-demographic characteristics of the older adults assessed were gender, age, marital status, and level of education. We asked them to state if they are male or female and we obtained the age by asking the older people to state their age as at the last birthday or to their year of birth. We recorded the age on a continuous level. It was however, categorised into (i) 60–65, (ii) 66–70, (iii) 71–75 and (iv) 76 and above. Similarly, three options were available for marital status; these include (i) married (ii) divorced and (iii) widowed.

Finally, for the level of education, we asked participants to indicate their highest level of education completed based on the following options. (i) no formal education (ii) primary (iii) secondary and (iv) post-secondary.

We assessed five illness characteristics: presence or absence of chronic illness, diagnosis, multimorbidity, the onset of illnesses, and prognosis. We asked for the presence or absence of a chronic illness with yes or no option, the specific diagnosis was assessed by asking the respondents to choose from the list of available options which contain hypertension, diabetes, chronic body pain, chronic cough, eye/ear disorder, and arthritis.

Furthermore, they were asked to choose whether there are other co-existing health conditions (multimorbidity). Respondents were also requested to state the onset of illness, options include (i) less than one year, (ii) 1–5 years, (iii), 6–10 years and (iv) more than ten years. Finally, we asked about changes (prognosis) they observed in the health conditions; the options include (i) improving, and (ii) deteriorating.

### Statistical analyses

Data were sorted, scored, and transformed to the WHOQOL-100 scale and then subjected to statistical analysis using SPSS 18.0. The score ranges from 0 to 100 and a higher the score indicates a better HRQoL. For analytical purposes, we used the overall (Total) HRQoL (comprising the summation of all 26 items) and created a dichotomous variable of good quality of life defined as a score of greater than the median, versus poor quality of life.

Descriptive statistics (frequency, percentage, mean, standard deviation) were used to describe the socio-demographic characteristics and illness characteristics of the older adults. Also, chi-square tests were used to assess the associations between HRQoL and the sociodemographic and illness characteristics of the older adults. Differences in the HRQoL of older adults with or without chronic illnesses was determined using Independent t-test. Furthermore, multiple regression analysis was used to identify the variables (socio-demographic and illness characteristics) that predict the HRQoL. We created dummies for gender (1 = woman, 0 = man), marital status (1 married, 0 = rest), chronic illness (1 = yes, 0 =no), diagnosis per illness (six) (1 = yes, 0 = no), onset (1 yes, 0 = no), prognosis (1 yes (deteriorating), 0 = rest). Age, level of education and multimorbidity were treated as continuous variables.

## Results

### Socio-demographic characteristics

Table 1 shows the socio-demographic characteristics of the respondents. The age ranged from 60 to 99, with a mean of 71.2 ± 4.73 years. More than half (54.6%) were within the age range of 60–65 years. Female accounted for 58.1% of the respondents, 72.7% of the respondents were married, while 34.2% had no formal education. 66.8% of the respondents reported a good quality of life. Increasing age, non-married status, and low-level of education were significantly associated with poor HRQoL (all p < 0.05). However, gender was not associated with HRQoL (see Table 1).

**Table 1:** Demographics characteristics and bivariate analysis of associations between overall health-related quality of life (HRQoL) and socio-demographic variables of the older adults

Variable	HRQ oL			
	Poor (n=125)	Good (n=252)	Total (n=377)	p-value
**Age Group**	n (%)	n (%)	N(%)	
**60–65**	40 (32)	166 (65.9)	206 (54.6)	< 0.001
**66–70**	43 (34.4)	63 (25.0)	106 (28.1)	
**71–75**	28 (22.4)	19 (7.5)	47 (12.5)	
**76 and above**	14 (11.2)	4 (1.6)	18 (4.8)	
**Gender**				
**Male**	49 (39.2)	109 (43.3)	158 (41.9)	0.453
**Female**	76 (60.8)	143 (56.7)	219 (58.1)	
**Marital status**				
**Married**	68 (54.4)	206 (81.7)	274 (72.7)	< 0.001
**Divorced**	7 (5.6)	7 (2.8)	14 (3.7)	
**Widowed**	50 (40)	41 (16.3)	91 (24.1)	
**Level of education**				
**No formal** **education**	54 (43.2)	75 (29.8)	129 (34.2)	0.015
**Primary**	29 (23.2)	71 (28.2)	100 (26.5)	
**Secondary**	23 (18.4)	48 (19.0)	71 (18.8)	
**Post-secondary**	19 (15.2)	60 (23.8)	79 (21.0)	

### Chronic illnesses

Table 2 presents the chronic illnesses of the respondents. More than half (53.3%) of the respondents had at least one chronic illness; the common conditions among the older adults included: hypertension (20.4%), diabetes (7.2%), chronic body pain (6.9%), chronic cough (2.4%) eye/ear disorder (5.6%), and arthritis (10.9%). More than a quarter (27%) had more than one illness, and the onset of illness showed that 21.2% have been living with the illness for 1–5 years; 40.8% claimed that the condition is improving. Furthermore, there is a significant association between HRQoL and the presence of chronic illness, type of diagnosis/condition, duration of illness, multimorbidity, and poor prognosis (all p < 0.001).

**Table 2:** Bivariate analysis of associations between overall health-related quality of life (HRQoL) and illness characteristics of the older adults

Variable	HRQoL			
	Poor (n=125)	Good (n=252)	n (%)	p-value
**Chronic illness**				
**Yes**	93 (74.4)	108 (42.9)	201 (53.3)	< 0.001
**No**	32 (25.6)	144 (57.1)	176 (46.7)	
**Diagnosis/condition**				
**None**	32 (25.6)	144 (57.1)	176 (46.7)	< 0.001
**Hypertension**	33 (26.4)	44 (17.5)	77 (20.4)	
**Diabetes**	9 (7.2)	18 (7.1)	27 (7.2)	
**Chronic body pain**	15 (12)	11 (4.4)	26 (6.9)	
**Chronic cough**	4 (3.2)	5 (2.0)	9 (2.4)	
**Eye/ear disorder**	8 (6.4)	13 (5.2)	21 (5.6)	
**Arthritis**	24 (19.2)	17 (6.7)	41 (10.9)	
**Multimorbidity**				
**None**	32 (25.6)	144 (57.1)	176 (46.7)	< 0.001
**1**	55 (44)	44 (17.5)	99 (26.3)	
**2**	33 (26.4)	26 (10.3)	59 (15.6)	
**3 or more**	25 (20)	18 (7.1)	43 (11.4)	
**Onset of illness**				
**None**	32 (25.6)	144 (57.1)	176 (46.7)	< 0.001
**Less than one year**	25 (20)	20 (7.9)	45 (11.9)	
**1–5 years**	28 (22.4)	52 (20.6)	80 (21.2)	
**6–10 years**	25 (20)	26 (10.3)	61 (13.5)	
**More than 10 years**	15 (12)	10 (4.0)	25 (6.6)	
**Prognosis**				
**N/A**	32 (25.6)	144 (57.1)	176 (46.7)	< 0.001
**Improving**	60 (48)	94 (37.3)	154 (40.8)	
**Deteriorating**	33 (26.4)	14 (5.6)	47 (12.5)	

### Differences in HRQoL between older adults with and without chronic illnesses

The mean scores in all the domains as well as the total WHOQOL-BREF score are presented in Table 3. The highest mean score was observed for the social relationships' domain (73.67± 9.34) and the lowest score in the physical health domain (64.06± 8.58) and the mean total HRQoL score 66.83±6.91. An independent t-test was run on the sample of 377 older adults comprising those with chronic illness (n=201) or those who have none (n=176) to determine if there were differences in their HRQoL (physical, psychological, social relationships and environmental domains and total HRQoL).

**Table 3:** Descriptive scores of the HRQol domains in relation to the presence or absence of chronic illness and the t-test results

	Chronic illness														
	All				None				Yes						
HRQoL Domain	Mean	SD	[95% C I]		Mean	SD	[95% Cl]		Mean	SD	[95% CI]		df	t	p
**Physical health**	64.1	8.58	34.3	85.7	65.64	7.17	64.64	66.63	62.25	9.65	60.82	63.69	375	-3.89	<0.001
**Psychological**	65.1	9.09	26.7	90.0	65.66	8.81	64.43	66.88	64.36	9.38	62.96	65.76	375	-1.38	0.168
**Social relationships**	73.7	9.34	33.3	100.0	76.02	7.84	74.93	77.11	70.98	10.18	69.47	72.5	375	-5.4	<0.001
**Environmental**	64.54	8.41	30.0	95.0	65.75	7.38	64.72	66.78	63.15	9.28	61.77	64.53	375	-3.03	0.003
**Overall**	53.5	5.53	30.2	71.5	54.61	4.63	53.97	55.26	52.15	6.16	51.23	53.07	375	-4.42	<0.001

The results showed that older adults with a chronic illness had statistically significantly lower HRQoL compared to those without a chronic illness in the following domains: physical health (with illness: 62.25± 9.65; without illness: 65.64±7.17, t =-3.89, p < 0.001), social relationships (without illness: 70.98±10.18; with illness: 76.02±7.84), t =-5.4150, p < 0.001); and environmental (with illness: 63.15±9.28; without illness: 65.75±7.38, t =-3.0274, p= 0.003); and the total HRQoL (with illness: 52.15±6.16; without illness: 54.61±4.63, t = -4.4185, p<0.001).

However, there was no statistically significant difference in the psychological domain of older adults with chronic illness (64.36±9.38) compared to those without chronic illness (65.66± 8.81), t =-1.3816, p= 0.168.

A multiple regression analysis was run to identify independent predictive variables for the total and domains of HRQoL (age, marital status, level of education, gender, presence or absence of chronic illness, diagnosis, multimorbidity, the onset of illness and prognosis of illness). The analyses showed that age was negatively associated with total HRQoL (β = -1.908, p = <0.001), physical health (β = -3.12, p <0.001), psychological (β = -1.618, p = 0.004, social relationships (β = -2.326, p =<0.001), and environmental (β = -2.472, P = <0.001). Similarly, marital status was negatively associated with total (β = -4.846, 1.479, p =0.001), physical health (β = 2.39, (p= 0.034), psychological (β = -5.356, p = 0.038), social relationships (β = -8.667, p =0.001), and environmental (β = -5.114, 0.029). Also, level of education was significantly associated with total (β = 0.674, p = 0.006), psychological (β =1.369, p = 0.001), and environmental (β = 1.319, p =0.001). Furthermore, chronic body pain is significantly associated with the environmental domain (β = 8.871, p = 0.038), while an onset of illness of 1–5 years was also negatively associated with the environmental domain (β = -6.218, p = 0.029). Finally, prognosis (improving) was negatively associated with social relationships (β=-11.521, p = 0.027) and environmental domains (β = -10.788, p = 0.022) (see Table 4).

**Table 4:** Multiple regressions model predicting the HRQoL of the older adults from their socio-demographic and illness characteristics

	HRQoL														
	Total (Overall)			Physical health			Psychological			Social relationships			Environmental		
Characteristic	B	SE	P	B	SE	P	B	SE	p	B	SE	P	B	SE	P
**Age**	-1.908	0.321	**<0.001**	-3.12	0.52	**<0.001**	-1.618	0.558	0.004	-2.326	0.558	**<0.001**	-2.472	0.505	**<0.001**
**Gender (women)**	-0.34	0.613	0.579	-0.77	0.99	0.438	1.289	1.067	0.228	-1.172	1.066	0.272	-1.071	0.965	0.268
**Marital status**	-4.846	1.479	**0.001**	-5.08	2.39	**0.034**	-5.356	2.575	**0.038**	-8.667	2.573	**0.001**	-5.114	2.33	**0.029**
**Level of Education**	0.674	0.243	**0.006**	0.25	0.39	0.526	1.369	0.423	**0.001**	0.419	0.423	0.322	1.319	0.383	**0.001**
**Chronic Illness**	8.495	5.136	0.099	-3.25	8.29	0.695	14.954	8.94	0.095	16.89	8.935	0.06	13.727	8.091	0.091
**Diagnosis**															
**Hypertension**	3.212	1.961	0.102	3.52	3.17	0.268	2.868	3.414	0.401	3.66	3.412	0.284	6.004	3.089	0.053
**Diabetes**	2.55	2.704	0.346	2.94	4.36	0.501	-0.642	4.706	0.892	1.558	4.703	0.741	8.871	4.259	**0.038**
**Chronic body pain**	0.385	3.221	0.905	1.25	5.2	0.81	-6.175	5.606	0.271	1.54	5.603	0.741	5.285	5.074	0.298
**Chronic cough**	-3.051	3.017	0.312	-0.71	4.87	0.884	-6.961	5.251	0.186	-5.984	5.248	0.741	-1.654	4.752	0.728
**Eye/Ear disorders**	-5.244	4.629	0.258	4.56	7.47	0.542	-13.59	8.057	0.093	-9	8.053	0.741	-8.152	7.292	0.264
**Multimorbidity**	-0.741	1.378	0.591	0.27	2.22	0.905	-2.895	2.398	0.228	-0.646	2.397	0.741	-0.431	2.17	0.843
**Onset**															
**Less than one year**	-3.004	2.311	0.194	-2.27	3.73	0.543	-4.208	4.023	0.296	-3.353	4.021	0.741	-5.085	3.641	0.163
**1–5 years**	-2.422	1.796	0.178	-0.54	2.9	0.852	-3.674	3.127	0.241	-1.653	3.125	0.741	-6.218	2.83	**0.029**
**6–10 years**	0.271	1.352	0.841	2.96	2.18	0.176	-1.583	2.354	0.502	1.358	2.353	0.741	-1.374	2.13	0.519
**More than 10 Years**															
**Prognosis**															
**Improving**	-4.921	2.984	0.1	3.33	4.82	0.49	-5.466	5.193	0.293	-11.52	5.191	**0.027**	-10.78	4.7	**0.022**
**Deteriorating**	-1.042	1.644	0.527	2.42	2.65	0.361	0.779	2.861	0.786	-5.133	2.859	0.073	-3.08	2.589	0.235
**Adj R-squared**		0.202			0.137			0.106			0.154			0.144	

## Discussion

The findings from this study revealed a high prevalence (53.3%) of chronic illnesses among the older adults in Osogbo, Osun State, Nigeria.

This is similar to previous study which revealed that 80% of the older adults have at least one chronic illness.^30^ A similar finding was also observed in a previous study among the Nigerian,^9^ Chinese,^31^ and American populations.^6^

This further buttressed the assertion that chronic conditions are a global issue that affects people's lives in both developed and developing countries.^32^

Additionally, findings from our study revealed that hypertension, diabetes, arthritis, and chronic body pain were the leading chronic illnesses among the older adults in this population. This finding is consistent with previous studies looking at chronic conditions among the older adults.^33^, ^34^

This study also showed that multimorbidity is prevalent (27.0%) among the older adults in Nigeria. This is supported by previous studies which stated that multimorbidity, is especially common in older people,^35^–^37^ and approximately two out of three individuals at retirement age suffer from at least two chronic diseases.^38^

The respondents rated their overall HRQoL as good and were satisfied with their present state of health. This supports the claim made by Netuveli and Blane 39 that ageing does not influence the quality of life negatively, after controlling for all other variables. The proposition made by previous authors might explain the good quality of life reported by the respondents.^39^,^40^ According to these authors with ageing from 50 years onward, the quality of life increases and peaks at 68 years before it starts to decline and reaches the same level as that of 50 years at 86 years. This finding is also in support of the expectation among the Yoruba people concerning illness perception and behaviour.

The Yoruba population, the dominant ethnic community in this study, usually expect that an individual confesses positively about his/ her state of health, even in the face of terrible and worsening health conditions. This is, however, contrary to WHO submission that quality of life decreased progressively as age increases..^25^

Our study also revealed that chronic illness is significantly associated with the HRQoL of the older adults. The HRQoL was lower in those with chronic illness compared with those without chronic illness. The most affected domains were physical health, social relationships, and environmental. This is in agreement with Lima and Barros^41^ who reported that there is a significant influence of illness on the HRQoL of the older adults. Similarly, Mwanyangala and Mayombana 42 documented that most frequent degenerative diseases such as cancer, hypertension, osteoporosis, and diabetes mellitus lead to reduced HRQoL.

Furthermore, Akinyemi and Owoaje 43 asserted that current illness is a good predictor of HRQoL and it is used to assess the impact of chronic illnesses such as diabetes and hypertension on the health status of the individual. Furthermore, the number of co-existing conditions also affect the HRQoL of a person. Thommasen and Zhang 14 also argued that the greater the number of illnesses, the worse the HRQoL in older age.

Furthermore, higher age, marital status (non-married), lower level of education, diagnosis (chronic body pain) onset (1–5 years) and prognosis (improving) were associated with a lower HRQoL of the older adults.

This finding confirms the multifactorial determinants of HRQoL which includes demographic, health, and social factors.^44^ Previous studies by Fajemilehin and Odebiyi 45 and Mugomeri and Chatanga 46 documented the importance of marital status to the quality of life in old age.

They stated that the presence or absence of a spouse would have influenced the health behaviour pattern and outcome of the chronic illness and subsequently affect their perception of their HRQoL. Besides, the study population holds marriage in high esteem, and a good marriage may be considered a great asset, thereby contributing to an individual's perception of quality of life. Netuveli and Blane 39 also asserted that marital status is associated with health and survival in old age.

Findings from this study also supports previous studies where it was observed that participants with low educational level usually report lower levels of HRQL than those with high educational level.^47^, ^48^ However, another study obtained a contrary report and it revealed that there was no statistically significant difference in HRQOL between patients with different educational level.^49^ Similarly, findings from this study have shown that age is associated with the HRQoL. This may not be unconnected with the fact that a large proportion of the older adults have at least one chronic illness and multiple morbidity even exists and this may even worsen HRQoL. This is however, contrary to Greenberger and Riddle 50 who reported that age did not have any effect on the HRQoL in their study among community dwelling elderly living in Tompkins County, NY.

The study findings are relevant considering the demographic transition in Nigeria together with the poor health system to manage the chronic illnesses. Results from this study have established the high prevalence of chronic illnesses in this population with a significant impact on the HRQoL of the older adults; this evidence is particularly relevant for policymakers. Thus, there is a need for regular health screening programs to be made available, accessible and affordable for the senior citizens to minimise disability, maintain independence at home, and prolonged survival as age-related conditions are preventable and reversible.

Due to the high prevalence of hypertension and other chronic illnesses among the Yorubas and by extension the Nigerian population, preventive health education, screening and early referral of identified cases should be encouraged to curb the prevalence and the possible complications that could arise from these health problems.

It is a common belief among the Yoruba population that reporting a suboptimal or poor quality of life or expressing dissatisfaction with one's state of health can lead to a decline in health status and general well-being. Further research should, therefore, objectively assess clinical health conditions and to determine its influence on their HRQoL. Besides, qualitative studies are encouraged to explore further the experience with chronic illness and the perception of HRQoL among the older population.

### Limitations of the study

Findings from this study were based on self-report from respondents rather than clinical examination. The possibility of non-disclosure of the true picture of the illness conditions and HRQoL of the respondents could not be ruled out. Also, the cross-sectional design of this study may not allow conclusions regarding causal relationships among the variables. In view of these, the generalisation of the findings should be handled with caution.

## Conclusion

This study showed that chronic illness is associated with reduced HRQoL among the older adults in Nigeria. Factors affecting the quality of life include age, marital status, the presence of chronic illness, diagnosis and multimorbidity.
